# The Other Face of Artesunate: Southern Drug to Treat Northern Diseases

**DOI:** 10.1016/j.ebiom.2014.11.017

**Published:** 2014-11-27

**Authors:** Stephane Picot

**Affiliations:** Malaria Research Unit, SMITH, ICBMS, UMR 5246 CNRS-INSA-CPE-UCBL1, University Lyon 1, France; Institut of Parasitology and Medical Mycology, Hospices Civils de Lyon, France

**Keywords:** Artesunate, Malaria, Cancer, Resistance

In this issue of *EBioMedicine*, Sanjeev Krishna and colleagues compared oral artesunate to placebo to treat colorectal cancer (Krishna et al., 2014). While the number of included patients was limited, they found a trend to higher recurrence-free survival probability at 42 months of follow-up after artesunate treatment. They failed to demonstrate that artesunate restored apoptosis in cancer cells, but they observed a decrease expression of Ki67 prognosis marker of colorectal cancer (Krishna et al., 2014). This double-blinded trial provides further evidence that artemisinin derivatives (ARTs) are not only antimalarial drugs. Do these data open new opportunities or drive new problems to malaria-endemic areas?

Evidence has emerged years ago that ARTs present a wide range of biological activities based on their alkylating capabilities, and may be effective against cancer cells by induction of cell apoptosis caused by reactive oxygen species production and angiogenesis inhibition (Hamacher-Brady et al., 2011). ARTs show potent in vitro activity for a variety of cancer cell lines (68 cell lines derived from solid tumors and 24 cell lines derived from hematological malignancies) at concentrations that are established or reachable in patients (Hooft van Huijsduijnen et al., 2013). Based on these in vitro results and the well-established safety of ARTs, evaluation of anticancer effect of ARTs in a clinical setting is warranted. One could expect such studies to be conducted in northern high-income countries free of malaria.

In southern low-income endemic areas, malaria still causes an unacceptable mortality rate and is responsible for more than half a million deaths each year, mainly in sub-Saharan Africa and in children under 5 years of age (O'Meara et al., 2010). Eliminating malaria requires preventing mosquito bites, accurate diagnosis, early treatment with a good drug at the right dose, and future mass vaccination. Considerable advancements have been made but malaria elimination still needs universal access to these strategies. Artemisinin and its derivatives have been the drugs of choice to treat malaria all over the world for more than a decade. These sesquiterpene lactones, including artesunate, artemether, arte-ether, dihydroartemisin, have demonstrated higher efficacies than all other known antimalarial drugs such as quinine. ARTs proved to have excellent tolerability in many controlled studies. While concerns have emerged in the early 2000s from animal studies when high doses were associated with damages to brainstem nuclei, the safety of short-term treatments with ARTs is well documented. ARTs saved millions of people from this devastating parasitic disease, and will continue to be used for that assignment.

The major drawback of ARTs is the potential of malaria parasites to escape from treatment after a few days, favoring the selection of drug-resistant parasites. This is the reason of the introduction of artemisinin-based combined therapies (ACTs) to treat malaria. These treatments are based on the association of ART that rapidly kills the parasites, and a partner drug with a prolonged half-life to complete the cure. The World Health Organization withdrawal of artemisinin monotherapy in malaria-endemic areas did not prevent the spread of drug resistance in South-East Asia (Ashley et al., 2014). While this resistance is still limited and efforts are made to contain its spread, ART use is put under surveillance (Talisuna et al., 2012). What will be the issues of using ARTs to treat other diseases? Is it only a geographical matter: cancer treatment in the north, malaria treatment in the south?

ARTs also showed μM activities against other parasites (*Schistosoma*, *Toxoplasma* and *Trypanosoma*), viruses (herpes virus, hepatitis B and hepatitis C viruses) and fungi (*Cryptococcus*), although clinical used in humans is mainly restricted to schistosomiasis so far. Schistosomiasis is the second most devastating parasitic disease in the world, leading to chronic and debilitating disease affecting 200 million people. Repeated prophylactic doses of ARTs dramatically reduce egg production and prevent 65–97% schistosomiasis cases. Toxoplasmosis is a serious concern during pregnancy and immunosuppression. In vitro, ARTs showed high efficacy against the parasite (Hencken et al., 2010), and mortality was reduced in a murine model of toxoplasmosis (Dunay et al., 2009). African and American trypanosomiases are severe diseases with a treatment hampered by high toxicity. Preliminary in vitro data showed that ARTs could be effective against these parasites (Nibret and Wink, 2010). Double-stranded DNA herpes viruses are very common and cause severe infections in immunocompromised patients. ARTs have shown 1–15 μM in vitro activity against cytomegalovirus and a rapid virus load decrease in infected patients after hematopoietic stem cell transplantation (Shapira et al., 2008). Taken altogether, these recent advances open new opportunities for a broader use of ARTs in tropical and non-tropical areas to treat several infectious diseases, besides malaria.

Considering the current study presented by Krishna et al. and other clinical studies, ARTs may also be used to treat different types of cancers in the future. One could speculate that these drugs may be mostly used in areas with a high-grade medical care, which should be malaria free. Thus the potential for these treatments to increase the spread of drug-resistant malaria parasite is considerably restricted by this epidemiological and economical difference. However, if the benefit of ARTs in cancer patients in confirmed, how will the prospect of using these drugs to treat cancer patients in malaria-endemic areas be addressed? How will we handle the access to artesunate treatment of colorectal cancer patients in countries such as Kenya, Thailand and Indonesia to limit the risk of selection of malaria-resistant parasites in asymptomatic carriers? Should we perform systematic high-sensitive malaria diagnosis in cancer patients before induction of treatment? Will it be necessary to add a partner drug in endemic areas and what will be the effects on cancer?

Artemisinins are demonstrating vast therapeutic potential for a large array of different diseases. Will the good news of a new usage for anticancer treatment open the Pandora's box for transmissible diseases? A controlled and sustainable use of new medication is required to limit the risks.

## Disclosure

The author declares no conflicts of interest.
